# Attributable fraction of tobacco smoking on selected cancer deaths in the past decade using mortality case-control study in Tianjin, China

**DOI:** 10.18332/tid/152203

**Published:** 2022-09-05

**Authors:** Wei Li, Xiaodan Xue, Dandan Li, Ying Zhang, Wenda Shen, Yi Pan, Dezheng Wang, Wenlong Zheng, Guohong Jiang

**Affiliations:** 1 Tianjin Centers for Disease Control and Prevention, Tianjin, China; 2 Public Health Department, Tianjin Medical University, Tianjin, China

**Keywords:** cancer, smoking attributed death, China, Asia, mortality case-control study

## Abstract

**INTRODUCTION:**

This study aims to estimate the impact of smoking-attributable mortality of selected cancers, in the period 2010–2019 in Tianjin, China.

**METHODS:**

A case-control study was conducted to assess the smoking-attributed major causes of cancer deaths. Unmatched multiple logistic regression was used to calculate mortality risk ratios.

**RESULTS:**

Smoking-attributable cancer deaths were 23709 (28.87%) among adult males and 8648 (13.37%) among adult females in the period 2010–2019 in Tianjin, China. Lung cancer remains the largest cause of smoking-attributable deaths; among men, the death rates were 49.06% of lung cancers, 27.55% of mouth, pharynx, larynx, or esophagus cancers, 13.56% of kidney and other urinary cancers, and 10.11% of liver cancers; among women the corresponding death rates were 31.56% of lung cancers and 10.59% of the mouth, pharynx, larynx, or esophagus cancer, 10.56% of bladder cancers. Smoking-attributed cancer deaths in men increased from 1817 in 2010 to 2695 in 2019; for women, the number remained stable at just over 800 per year during the past decade.

**CONCLUSIONS:**

At least one in three cancer deaths in men and one in six in women would be potentially preventable through appropriate control of tobacco smoking in Tianjin, China. Effective control programs against tobacco smoking should be further implemented.

## INTRODUCTION

Cancer has become the largest public health problem in the world^1^, and the prevention and treatment of cancer in China has become an important goal. Asia has a high cancer incidence and mortality, while China has the highest incidence in Asia. The results of the cancer survey in China from 2004 to 2005 showed that the cancer mortality rate increased by 83.1% compared with that in the mid 1970s, and 22.5% higher than that in the early 1990s^2^. According to the Statistics of the World Cancer Report^3^, in 2012, China accounted for 20% of the world’s new cancer cases and 25% of the deaths.

Smoking is by far the most important risk factor of cancer that can be modified at the individual lifestyle level. More than one in four cancer deaths (29%) in men and one in five cancer deaths (20%) in women can be attributed to smoking^4^. However, the values vary from country to country. Knowledge of the cancer deaths caused by smoking in each country or region has a significant impact on the disease control and prevention.

Tianjin is one of the largest and most developed cities in China. It has a more sophisticated and reliable system of death registration than most cities in China and in other low- and middle-income countries. The annual death reporting rate in Tianjin has been maintained at 6.5–7.0‰ for several years, reaching the international gold standard, and the underreporting rate, information missing rate, rate of coding error and unknown cause of death diagnosis are decreasing year by year. Tianjin All Causes of Death Registration System (CDRS) was established in 1984, which now covers the entire population of approximately 10 million (from 9.85 million in 2010 to 11.08 million in 2019) with 40% urban and 60% rural residents^5^. Through this system, all causes of death should be reported, and all death certificates entered into the database must be completed by physicians in hospitals or community health service centers.

Starting at the end of year 2009, Tianjin Centers for Disease Control and Prevention (TJCDC) has been collecting information routinely on smoking of the deceased in the death certificate. This is the first and only death registration system in China doing so. Tianjin, being the second to include smoking in death registration in the world after South Africa, collects more information on smoking than South Africa. Tianjin developed mortality case-control (MCC) studies to use the information collected from CDRS since 2010^6^.

The purpose of the current study was to perform an evidence-based assessment of the burden of selected smoking-attributable cancers on mortality in the Tianjin, China.

## METHODS

### Data source

We got the smoking information and cause of death from the CDRS. Each death certificate has approximately 50 data fields, including age, sex, education level, and cause of death, as well as the home address for classifying area of residence as urban or rural. The 3 questions about smoking on the death certificate were: 1) smoking status, i.e. current, former and never smoker; 2) number of cigarettes smoked per day; and 3) number of years of smoking. The information was obtained by doctors through asking relatives that lived with the deceased and by consulting medical records. When information was not available, the doctors would leave the answer space as blank.

### Definition of case and control

We included deaths of people aged ≥35 years between 2010 and 2019. We excluded deaths at age <35 years because smoking was expected to cause very few deaths at a young age, as death caused by smoking would be less reliable. We also excluded deaths without smoking information. We followed the method of Sitas et al.^7^, and the definition of cases and controls were according to the American Cancer Society’s ‘A Report of the Surgeon General’^8^. Cases were deaths from 9 selected groups of cancers that are causally or strongly associated with smoking including lung, the mouth, pharynx, larynx, or esophagus, stomach, liver, pancreas, colorectal, kidney and other urinary, bladder, and myeloid leukemia. Controls were deaths from all other specified diseases that were not confirmed to be caused by or were not strongly associated with smoking. Such as infectious and parasitic diseases, diseases of the nervous system, digestive diseases, genitourinary diseases, nutritional deficiencies, musculoskeletal diseases, skin infections and disorders, benign neoplasm, miscellaneous and minor conditions, acute rheumatic fever or chronic rheumatic heart disease, congenital anomalies, oral diseases, and diseases of the sense organs. Ill-defined and unknown causes were also excluded. The cases were current and former smokers and controls were never smokers. We used the term ‘ever smoker’ to mean ‘former’ or ‘current’ smoker in this study.

### Ethics statement

This study was implemented as a register-based study based on anonymous data at Tianjin Centers for Disease Control and Prevention and was approved by the Tianjin Centers for Disease Control and Prevention Ethics Committee. Register-based studies on anonymous data do not require participant informed consent in Tianjin.

### Relative risk of tobacco smoking and estimation of population attributed fraction (PAF)

Unmatched multiple logistic regression was used to calculate risk ratios (RR) or mortality rate ratio of all-cause and cause-specific death in ever smokers versus never smokers, adjusted by 5-year age group, education level (none which means illiteracy, primary which included education level is high school and below, higher which included education level higher than high school, and do not know), marital status (never, widowed, divorced, married or living as married, do not know), and year of death in the period 2010–2019.

If D is the total number of deaths (from a particular disease) in smokers, then the number of deaths attributed to smoking, Ds, can be calculated separately for men and women as: Ds = D (1 - 1/RR), where RR is risk ratio (to estimate relative risk), where Ds is positive, Ds divided by the total number deaths in the whole population gives the PAF of smoking^9^. Where Ds are negative, the fraction could be due to the ‘protective’ effect of smoking. Attributable death numbers for groups of diseases (and for overall mortality) were derived from the sum of the number of deaths from each disease within the group.

Men and women were analyzed separately. Statistical tests and 2-sided 95% confidence intervals (CIs) were based on changes in log-likelihood. When the 95% CI for the relative risk in men did not overlap with that in women, the sex difference was significant at p<0.05. Otherwise, the interaction of sex and smoking was tested by adding an interaction term in the model. All analyses were performed with SPSS 24.0 (SPSS Inc).

### Sensitivity analysis for the estimation of PAF of tobacco smoking

To account for the uncertainty in PAF estimation arising from the estimation of RRs for each cancer site, a sensitivity analysis was performed under alternative scenarios using the lower and upper limits of the 95% CIs of RR estimates.

## RESULTS

### Relative risk of tobacco smoking

Among all cancer sites reviewed in this study, the total RR for ever smokers was 1.861 and 2.380 in men and women, respectively (Table 1). Top three RRs among ever smoker men were lung cancer (RR=3.06; 95% CI: 2.96–3.17), the mouth, pharynx, larynx, or esophagus cancers (RR=1.80; 95% CI: 1.70–1.90), and kidney and other urinary cancers (RR=1.33; 95% CI: 1.21–1.47). Lung cancer (RR=3.87; 95% CI: 3.70–4.05), the mouth, pharynx, larynx, or esophagus cancers (RR=1.71; 95% CI: 1.54–1.90) and bladder cancer (RR=1.68; 95% CI: 1.42–1.99) were the top three among women. The RRs for ever smokers ranged from 1.08 to 3.87 for cancer mortality, except for a few cases where the RR was estimated <1 with insignificant p-values (Table 1).

**Table 1:** Mortality risk ratio (RR) at age ≥35 years, selected cancer causes, ever smokers versus never smokers, Tianjin, China, 2010–2019

*Site (ICD-10 Code)*	*2010*		*2014*		*2019*		*Total*	
	*RR*	*95% CI*	*RR*	*95% CI*	*RR*	*95% CI*	*RR*	*95 % CI*
**Men**								
Lung (C33-4)	3.013	2.644–3.434	3.166	2.833–3.537	3.162	2.852–3.505	3.061	2.956–3.170
Mouth, pharynx, larynx, or esophagus (C00-15, C32)	1.365	1.111–1.677	1.905	1.589–2.284	1.904	1.604–2.259	1.798	1.698–1.904
Stomach (C16)	1.070	0.895–1.280	1.202	1.024–1.412	1.131	0.966–1.324	1.189	1.129–1.251
Liver (C22)	1.165	0.996–1.363	1.339	1.157–1.549	1.214	1.053–1.400	1.227	1.172–1.284
Pancreas (C25)	1.036	0.806–1.332	1.436	1.158–1.780	1.120	0.926–1.354	1.189	1.114–1.270
Colorectal (C18-20)	0.987	0.801–1.217	1.213	1.021–1.440	1.023	0.879–1.191	1.078	1.022–1.138
Kidney and other urinary (C64-6, C68)	1.112	0.762–1.624	1.755	1.288–2.392	1.237	0.950–1.611	1.333	1.210–1.468
Bladder (C67)	1.580	1.134–2.201	1.309	0.998–1.718	1.419	1.112–1.810	1.329	1.220–1.447
Myeloid leukemia (C92, C93.0, C94.0, C94.2, C94.4-5)	1.062	0.466–2.422	0.821	0.416–1.618	0.794	0.431–1.462	1.011	0.820–1.248
Total	1.745	1.563–1.948	1.987	1.806–2.186	1.846	1.693–2.013	1.861	1.807–1.918
**Women**								
Lung (C33-4)	4.046	3.483–4.700	3.699	3.200–4.276	3.964	3.471–4.529	3.870	3.703–4.045
Mouth, pharynx, larynx, or esophagus (C00-15, C32)	1.890	1.359–2.630	1.464	1.033–2.076	2.032	1.458–2.831	1.708	1.536–1.899
Stomach (C16)	1.135	0.842–1.528	1.048	0.770–1.425	1.161	0.861–1.566	1.090	0.990–1.200
Liver (C22)	1.257	0.991–1.595	1.001	0.759–1.319	1.422	1.095–1.846	1.215	1.122–1.316
Pancreas (C25)	1.475	1.084–2.007	1.218	0.890–1.668	1.343	1.018–1.771	1.161	1.054–1.279
Colorectal (C18-20)	0.970	0.717–1.312	0.899	0.677–1.194	1.138	0.892–1.451	0.975	0.895–1.061
Kidney and other urinary (C64-6, C68)	0.680	0.327–1.415	1.196	0.688–2.078	1.028	0.619–1.706	1.121	0.940–1.337
Bladder (C67)	1.513	0.810–2.823	1.881	1.085–3.261	1.647	0.997–2.719	1.679	1.416–1.991
Myeloid leukemia (C92, C93.0, C94.0, C94.2, C94.4-5)	0.333	0.042–2.659	-	-	0.797	0.187–3.396	0.785	0.484–1.271
Cervix (C53)	1.009	0.503–2.024	1.049	0.611–1.802	1.192	0.725–1.960	1.107	0.937–1.309
Total	2.402	2.096–2.752	2.296	2.007–2.626	2.445	2.168–2.757	2.380	2.285–2.478

### Smoking-attributable cancer deaths by age and cancer type

Tobacco smoking was responsible for 23709 (28.87%) cancer deaths among adult men and 8648 (17.37%) cancer deaths among adult women between 2010 and 2019 in Tianjin, China (Table 2). As expected, lung cancer comprised the greatest proportion of all smoking-related cancer deaths both in men and women.

**Table 2:** Number of smoking-attributable cancer deaths and attributable fraction by site, Tianjin, China, 2010–2019

*Site (ICD-10 Code)*	*2010*		*2014*		*2019*		*Total*	
	*Current and ever %*	*Deaths attributed to smoking n (%)*	*Current and ever %*	*Deaths attributed to smoking n (%)*	*Current and ever %*	*Deaths attributed to smoking n (%)*	*Current and ever %*	*Deaths attributed to smoking n (%)*
**Men**								
Lung (C33-4)	75.32	1549 (50.32)	77.82	1886 (48.91)	74.46	2183 (50.91)	72.87	18705 (49.06)
Mouth, pharynx, larynx, or esophagus (C00-15, C32)	58.43	80 (15.62)	60.86	189 (29.00)	65.94	243 (31.31)	62.07	1802 (27.55)
Stomach (C16)	51.07	25 (3.34)	48.78	71 (8.20)	51.46	51 (5.96)	50.82	674 (8.08)
Liver (C22)	56.99	96 (8.07)	54.09	169 (13.69)	56.03	120 (9.88)	54.65	1282 (10.11)
Pancreas (C25)	51.27	6 (1.78)	53.70	68 (16.30)	52.37	31 (5.61)	52.60	380 (8.36)
Colorectal (C18-20)	48.78	-3 (-0.64)	48.02	60 (8.43)	48.03	10 (1.08)	47.64	250 (3.45)
Kidney and other urinary (C64-6, C68)	53.17	7 (5.36)	58.38	46 (25.11)	53.91	26 (10.33)	54.26	253 (13.56)
Bladder (C67)	58.33	36 (21.41)	47.92	27 (11.31)	53.29	48 (15.74)	49.90	302 (12.35)
Myeloid leukemia (C92, C93.0, C94.0, C94.2, C94.4-5)	45.83	1 (2.68)	40.54	-3 (-8.84)	43.18	-5 (-11.20)	46.70	2 (0.51)
Total	63.96	1817 (27.31)	61.58	2506 (30.59)	63.77	2695 (29.22)	62.39	23709 (28.87)
**Women**								
Lung (C33-4)	48.62	807 (36.60)	41.74	804 (30.46)	39.58	754 (29.60)	42.56	8035 (31.56)
Mouth, pharynx, larynx, or esophagus (C00-15, C32)	31.55	31 (14.86)	22.73	16 (7.20)	26.05	28 (13.23)	25.54	229 (10.59)
Stomach (C16)	19.55	8 (2.33)	15.31	3 (0.70)	14.15	8 (1.96)	15.33	50 (1.27)
Liver (C22)	21.33	26 (4.36)	14.95	0 (0.01)	17.10	25 (5.08)	17.60	171 (3.11)
Pancreas (C25)	23.71	22 (7.64)	16.71	11 (2.99)	16.13	19 (4.12)	16.15	84 (2.24)
Colorectal (C18-20)	16.41	-2 (-0.51)	14.00	-8 (-1.57)	14.67	12 (1.78)	14.63	-20 (-0.38)
Kidney and other urinary (C64-6, C68)	14.29	-4 (-6.72)	16.50	3 (2.70)	14.29	1 (0.39)	16.52	18 (1.78)
Bladder (C67)	27.27	5 (9.25)	26.32	9 (12.33)	25.00	9 (9.82)	26.12	80 (10.56)
Myeloid leukemia (C92, C93.0, C94.0, C94.2, C94.4-5)	5.88	-2 (-11.78)	0.00	0 (0.0)	5.00	0 (0.00)	8.76	0 (0.00)
Cervix (C53)	14.29	0 (0.13)	11.04	1 (0.52)	10.50	3 (1.69)	11.09	17 (1.07)
Total	35.33	876 (20.62)	29.61	831 (16.72)	27.58	853 (16.30)	29.96	8648 (17.37)

In men, nearly 50% of lung cancer deaths, 27.55% of the mouth, pharynx, larynx, or esophagus cancer deaths, 10.11% of liver, 8.08% of stomach, 8.36% of pancreas, 13.56% of kidney and 12.35% of bladder cancer deaths were attributable to tobacco smoking. In women, however, smoking-attributable lung cancer deaths were 31.56% of the total lung cancer deaths; 10.59% of the mouth, pharynx, larynx, or esophagus cancer deaths; and 10.11% of liver, 8.08% of stomach, 10.56% of bladder cancer deaths were attributable to tobacco smoking. In women, the smoking-attributable deaths in 2010, 2014 and 2019 were 20.62%, 16.72% and 16.30%, respectively, indicating a decreasing trend, not found in men, where the corresponding smoking-attributable deaths were 27.31%, 30.59% and 29.22 % (Table 2).

The smoking rate of men in the study (who died between 2010 and 2019) remained stable for 10 years (average: 62.39%), while women showed a downward trend (from 35.33% to 27.58%).

**Figure 1:**
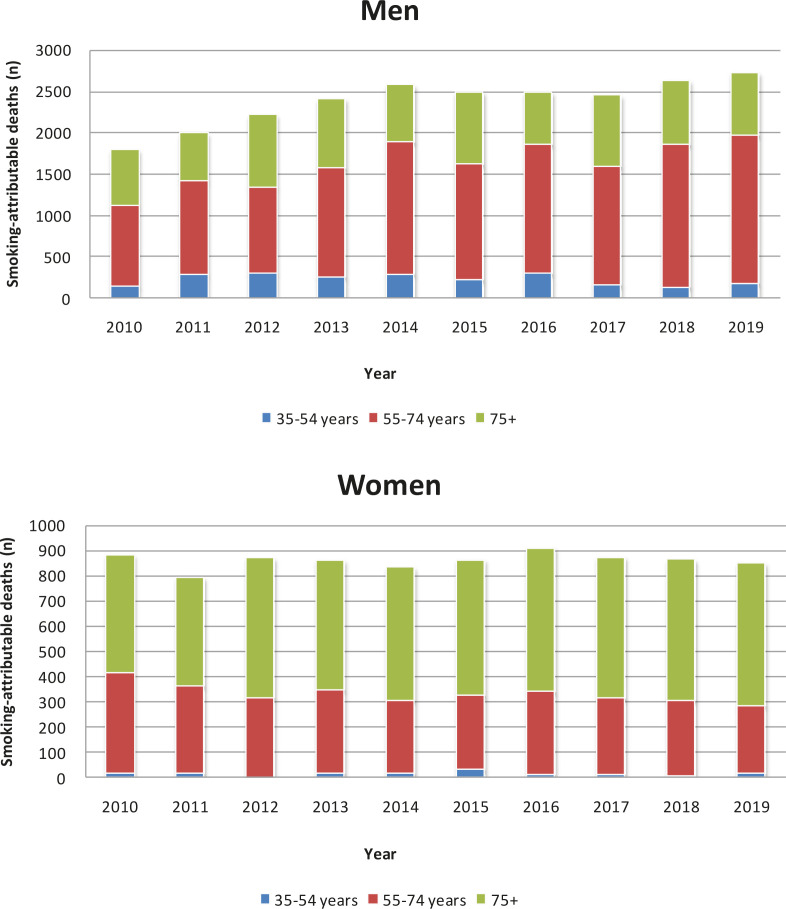
Smoking-attributable cancer deaths by age group, males and females, Tianjin, China, 2010–2019

Despite the population growth and ageing during the study period, the annual number of smoking-attributable cancer deaths among women has remained relatively constant, at around 800 each year since 2010 (Figure 1). The number of smoking-attributable cancer deaths in women aged 55–74 years decreased by 31.81%, and increase by 20.77% in those aged ≥75 years, between 2010 and 2019 (Table 3). The annual number of smoking-attributable cancer deaths among men increased from 1822 to 2684 (increase by 47.3%) from 2010 and 2019 (Figure 1 and Table 3). The number of smoking-attributable cancer deaths in men aged 35–54, 55–74, ≥75 years increased by 27.40%, 82.31% and 11.56%, respectively, between 2010 and 2019 (Table 3). The greatest increase in the annual number of smoking-attributable cancer deaths was in men aged 55–74 years, from 978 in 2010 to 1786 in 2019.

**Table 3:** Number of smoking-attributable cancer deaths and attributable fraction by age, 2010–2019

*Age (years)*	*2010*		*2014*		*2019*		*Total*	
	*Current and ever %*	*Deaths attributed to smoking n (%)*	*Current and ever %*	*Deaths attributed to smoking n (%)*	*Current and ever %*	*Deaths attributed to smoking n (%)*	*Current and ever %*	*Deaths attributed to smoking n (%)*
**Men**								
35–54	68.94	146 (17.41)	67.31	296 (35.75)	66.18	186 (30.08)	66.22	2331 (28.55)
55–74	67.03	978 (29.54)	66.17	1591 (36.59)	69.83	1786 (33.69)	67.09	13941 (31.80)
≥75	58.24	675 (26.93)	53.40	689 (22.85)	53.58	753 (22.82)	54.52	7580 (25.16)
Total	63.96	1822 (27.39)	61.58	2514 (30.68)	63.77	2684 (29.11)	62.39	23783 (28.96)
**Women**								
35–54	9.77	19 (4.65)	6.88	15 (3.76)	8.22	16 (5.29)	8.42	146 (3.65)
55–74	34.92	396 (20.71)	24.90	292 (13.53)	20.86	270 (11.57)	25.51	3214 (14.77)
≥75	41.01	467 (24.11)	37.68	527 (21.85)	35.86	564 (21.71)	37.56	5306 (22.07)
Total	35.33	877 (20.65)	29.61	835 (16.78)	27.58	852 (16.28)	29.96	8648 (17.37)

### Sensitivity analysis for the estimation of PAF of tobacco smoking

**Figure 2:**
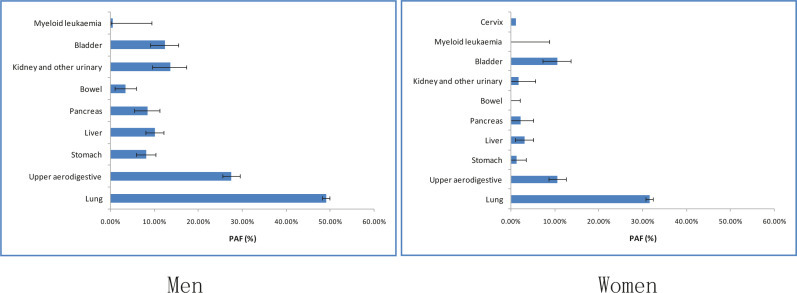
Sensitivity analysis of the PAF for ever smokers using the lower and upper limits of 95% confidence interval for relative risks

To account for the uncertainty in PAF estimation arising from the estimation of RRs for each cancer site, a sensitivity analysis was performed under alternative scenarios using the lower and upper limits of the 95% CIs of RR estimates (Figure 2).

## DISCUSSION

Our study provides a systematic assessment of the burden of smoking on selected cancers in Tianjin, China, in the period 2010–2019. The nationwide prospective cohort study in China gave RRs of lung cancer among urban smoking men as 2.98 (95% CI: 2.66–3.33) and 2.32 (95% CI: 2.08–2.59) in 2010 and 1995, respectively, and showed an increasing trend. In our study, the RR for ever smoker deaths from lung cancer in men was 3.013, 3.166 and 3.162 in 2010, 2014 and 2019, respectively. Among women, RR of ever smokers was 2.56 (95% CI: 2.02–3.26) in lung cancer women in the study of Chen et al.^10^ (about 2010). In our study, the RR of lung cancer was 4.046 (95% CI: 3.483–4.700) in 2010, higher than their result. It may be related to the high smoking prevalence of women in Tianjin. The RR for women deaths from lung cancer in Tianjin was similar to those of Japan and Korea, at 3.6 and 3.2, respectively^11^. But the results were much lower than in France and United Kingdom.

Overall, 24.53% selected cancer deaths were attributable to tobacco smoking in Tianjin. There was a large discrepancy between men and women in the PAF estimates of cancer mortality (28.87% vs 17.37%), which was a little lower than previous reports in men in Korea (32.9%), Japan (34.4%), and France (33.4%), and much higher than in Korea (5.2%), Japan (6.2%), and France (9.6%)^11-13^ for women. In the United Kingdom, PAF was 23.0% and 15.6% in men and women, respectively. Our results suggest that women have the highest percentage of all-cause and lung cancer deaths attributable to smoking in Tianjin. This may be because of their high smoking prevalence. The First National Smoking Prevalence Survey in 1984 showed that the smoking prevalence was 7.04% in women in China, while the prevalence of Tianjin women was 27.4%, which is the highest^14^. Also, we found that the number of smoking-attributable cancer deaths increased only in people aged ≥75 years (from 467 to 564) between 2010 and 2019. That was also because the smoking prevalence of the women born before 1935 was higher than for women born after 1935.

These results support the necessity of country and region evaluation of the PAF because even though the overall PAF appears to be the same, the exposure prevalence and the RRs can be different across countries or districts, therefore, the prevention strategy in each country or district should be different. It is very important to monitor and calculate RR and PAF at the country and regional level for formulating targeted prevention and control strategies.

Due to the significant decrease of smoking rate in women^15,16^, smoking-attributable lung cancer deaths in women decreased from 36.6% to 29.6% during the study period, but remained stable in men. Since the majority of lung cancers were attributed to smoking, changes in smoking prevalence are the key driver of lung cancer mortality trends. In particular, 49.06% of all lung cancer deaths in Tianjin more among men aged >35 years could have been prevented if no man smoked in Tianjin; the percentage was 31.56% in women.

The MCC study developed in the Tianjin design can be a quick, efficient, and reliable method to assess and monitor mortality risks of smoking, and it can be promoted in other cities or countries with a reliable system of death certification and good quality control measures like the Tianjin CDRS. Use of RRs from the MCC study in Tianjin can measure more accurately smoking-attributable deaths than general RRs.

Our findings highlight the high risk of cancer deaths, mainly from lung cancer and mouth, pharynx, larynx, or esophagus cancers, in men and women ever smokers and particularly the high proportion of deaths attributable to smoking in women in Tianjin, which had the highest smoking prevalence among women around the 1980s in China. Strong tobacco control measures are needed to motivate a large proportion of smokers, including women smokers, to stop smoking. And also, special and urgent warnings and tobacco control campaigns are needed to prevent the increase in smoking in young women. Our results also highlight that in countries, regions, or cities, with a reliable system of death certification, the mortality case-control study design using routinely collected smoking data from death certificates can be used to rapidly and periodically assess the mortality risks of smoking and evaluate the effects of tobacco control measures at different stages of the tobacco use epidemic.

### Strengths and limitations

This study is China’s first mortality case-control study based on smoking data from death registration. Lung cancer was the main cause (half in men and two-thirds in women) of smoking-induced deaths. The smoking-attributed fractions of all-cause and lung cancer deaths in women were the greatest probably because of the high smoking prevalence among woman in the city around the 1980s. The mortality case-control study design can be used to rapidly and periodically assess the mortality risks of smoking and evaluate the effects of tobacco control measures.

Our study has some limitations. First, the accuracy of the smoking status review in mortality case-control studies may affect the results. In order to prove the accuracy of the data, we conducted a call-back survey on smoking status, and the accuracy was higher than 75%. The relevant results have been published^5^. Second, the selected cancer types in our study were limited. We should add other types of cancer such as breast cancer in a future study. Third, we did not contain the harm of exposure to secondhand smoke. This may underestimate the impact of tobacco on cancer deaths.

### Implications

Our study provided a systematic assessment of the burden of smoking-related cancer by mortality case-control study in Tianjin, China in the period 2010–2019. RR for ever smokers for death from lung cancer in men was 3.061 (95% CI: 2.956–3.170), similar to the nationwide prospective cohort studies result (RR=2.98; 95% CI: 2.66–3.33). But the RR for women was higher in our study (RR=3.870; 95% CI: 3.703–4.045 vs RR=2.56; 95% CI: 2.02–3.26) than for the whole of China, but similar to those of Japan and Korea. Overall, 32357 of 131919 (24.53%) cancer deaths were attributable to tobacco smoking in Tianjin. There was a large discrepancy between men and women in the PAF estimates of cancer mortality (28.87% vs 17.37%). These results indicate that it is very important to monitor and calculate RR and PAF at country and region level for formulating targeted prevention and control strategies.

## CONCLUSIONS

While the smoking prevalence in adult males has been decreasing in Tianjin, China, it remains high (more than 25% in those aged ≥15 years)^17^. Because Tianjin is quickly approaching the status of an aged society, the number of cancer cases and deaths are expected to increase in the future. Approximately one in three selected cancer deaths in men and one in six selected cancer deaths in women would be potentially preventable through appropriate control of tobacco smoking in Tianjin. Effective tobacco control programs should be further developed and implemented in Tianjin to reduce the smoking-related cancer burden.

## Data Availability

The data supporting this research cannot be made available for privacy or other reasons.
